# A statistical approach to quantitative data validation focused on the assessment of students’ perceptions about biotechnology

**DOI:** 10.1186/2193-1801-2-496

**Published:** 2013-10-01

**Authors:** Maria João Fonseca, Patrício Costa, Leonor Lencastre, Fernando Tavares

**Affiliations:** CIBIO - Centro de Investigação em Biodiversidade e Recursos Genéticos/InBIO Laboratório Associado, Universidade do Porto, Campus Agrário de Vairão, Rua Padre Armando Quintas, 4485-661 Vairão, Portugal; Faculdade de Ciências, Departamento de Biologia, Universidade do Porto, Porto, Portugal; IBMC - Instituto de Biologia Molecular e Celular, Universidade do Porto, Porto, Portugal; Life and Health Sciences Research Institute (ICVS), School of Health Sciences, University of Minho, Braga, Portugal; PT Government Associate Laboratory, ICVS/3B’s, Braga, Portugal; Faculdade de Psicologia e de Ciências da Educação, Universidade do Porto, Braga, Portugal

**Keywords:** Biotechnology education, Exploratory factor analysis, Elementary and high school, Psychometric analysis, Questionnaire validation, Reliability

## Abstract

Student awareness levels are frequently used to evaluate the effectiveness of educational policies to promote scientific literacy. Over the last years several studies have been developed to assess students’ perceptions towards science and technology, which usually rely on quantitative methods to achieve broad characterizations, and obtain quantifiable and comparable data. Although the usefulness of this information depends on its validity and reliability, validation is frequently neglected by researchers with limited background in statistics. In this context, we propose a guideline to implement a statistical approach to questionnaire validation, combining exploratory factor analysis and reliability analysis. The work focuses on the psychometric analysis of data provided by a questionnaire assessing 1196 elementary and high school students’ perceptions about biotechnology. Procedural guidelines to enhance the efficiency of quantitative inquiry surveys are given, by discussing essential methodological aspects and relevant criteria to integrate theory into practice.

**Electronic supplementary material:**

The online version of this article (doi:10.1186/2193-1801-2-496) contains supplementary material, which is available to authorized users.

## Introduction

It is widely acknowledged that while case-study methodologies are particularly appropriate for detailed studies, survey-based approaches can provide considerable amounts of data that are usually easier to process and suitable for prediction and generalization, in comparatively shorter periods of time (Black 1999 Oppenheim 1992). These features underlie the reasons why in large scale educational observatories such as the ROSE, PISA and TIMSS surveys, quantitative assessment is favoured. Among the various inquiry methods available, quantitative approaches such as questionnaire-based surveys allow broad characterizations of target populations (Black 1999 Oppenheim 1992). However, these instruments hold limitations, mainly pertaining to biases introduced by the respondents’ subjective interpretations and the researcher’s expectations (Black 1999). Hence, questionnaires must be designed and administered following adequate procedures to optimize the validity and reliability of the results provided. Questionnaire validation, namely by adopting an integrated approach combining pilot study with psychometric analysis, allows improving the instrument’s design and addressing ambiguities that can compromise the quality of the data gathered (Black 1999 Fabrigar et al. 1999 Oppenheim 1992). Still, there are many studies in which these procedures are insufficiently or inappropriately reported (Blalock et al. 2008). For instance, Blalock et al. (2008) analyzed 150 peer-reviewed articles published between 1935 and 2005 focusing on the measurement of students’ attitudes towards science and verified that, from the 66 resulting instruments, 42% were missing evidence of psychometric soundness. This may result from the seeming complexity of the available validation methods and from the erroneous perception that they are inefficient considering the extra time and effort demanded for their implementation Dörnyei (2002). However, if research designs overlook validation procedures, the resulting data will not allow for sound interpretation, reproducibility and comparison. Therefore, validation is a crucial topic in science education research.

This study proposes a guideline for the improvement of the quality of quantitative data by discussing a statistical approach to psychometric analysis, combining exploratory factor analysis and reliability analysis. The work presented focuses on the large scale evaluation of a multidimensional questionnaire, developed and validated to assess elementary and high school students’ perceptions about biotechnology.

### Exploratory factor analysis

Exploratory factor analysis (EFA) is an exploratory method to probe data variations in search for a more limited set of variables, or factors that can explain the variability observed for the variables measured, that has become a frequently used statistical technique in psychometric analysis (Costello and Osborne 2005Fabrigar et al. 1999Henson and Roberts 2006). Through the combination of the predicted variables within the components identified, EFA allows reducing the total number of variables to process and, most importantly, assessing construct validity (Hayton et al. 2004) by enabling the quantification of the extent to which the items measure the intended constructs (Groth-Marnat 2009). Nevertheless, the empirically endorsed good practices in EFA require making a considerable amount of decisions based upon contextual parameters rather than on clearly predetermined criteria (Child 2006Costello and Osborne 2005Hogarty et al. 2005). Amongst such decisions, the ones that most frequently concern researchers deal with the size of the sample used and the number of factors and items to retain (Costello and Osborne 2005).

## Context of the study

With the recognition of the range and depth of biotechnology’s social repercussions, the concerns about the public’s understanding of biotechnology applications have fostered an increasing curricular coverage of biotechnology topics, and the development of numerous programs and resources to promote students’ literacy (Dawson and Soames 2006; Sáez et al. , Sáez et al. Sáez et al. 2008). Across numerous countries, including Australia, The Netherlands, Slovenia and Turkey, the efficiency of this investment in biotechnology education has been mainly evaluated using quantitative instruments designed to assess students’ knowledge and attitudes towards (Dawson and Soames 2006Klop and Severiens 2007Prokop et al. 2007; Uşak et al. , Uşak et al. Uşak et al. 2009). However, more commonly that what could be expected, the instruments used are not psychometrically adequate, and the reported findings are based on data obtained using questionnaires that have not been properly validated (Erdogan et al. 2009). Besides affecting the validity of eventual comparisons established according to the indicators conveyed in these studies, this also compromises the reliability of the diagnostic assays, which ultimately impacts the success of interventions designed accordingly. These implications emphasize the need to further extend the array of studies focusing on students’ perceptions of biotechnology using valid measurement instruments, and to assist researchers in making sense of the psychometric analysis methods available.

The integration of information obtained by measuring different elements that affect opinions and behaviours regarding biotechnology, such as knowledge, attitudes, interest, and importance attributed to it can contribute to a more thorough understanding of the factors that mediate students’ perceptions. However, most of the studies addressing students’ perceptions about biotechnology have generally covered knowledge and attitudes (Dawson 2007Klop and Severiens 2007Prokop et al. 2007; Uşak et al. , Uşak et al. Uşak et al. 2009), and a more limited number has assessed student interest about this socio-scientific issue (Kidman 2008; Lamanauskas and Makarskaitė-Petkevičienė , Lamanauskas and Makarskaitė-Petkevičienė Lamanauskas and Makarskaitė-Petkevičienė 2008). Accordingly, the questionnaires that have been made available focus on the measurement of discrete elements. Furthermore, these instruments often lack empirical support of validity and reliability. So far, only a limited number of studies, as for instance the ones by Klop and Severiens (2007) and Erdogan et al. 2009have clearly evidenced concerns with the psychometric soundness of the instruments used. If the existent questionnaires are not utterly appropriate to address the specificities of the target population or account for the entire topics one intends to investigate, it becomes necessary to develop novel instruments that must be thoroughly validated. In fact, validation must always be incorporated in a study’s design, as these procedures report to specific settings and populations (Oppenheim 1992). Therefore, aiming to obtain a broader and articulated appraisal of elements that mediate students’ perceptions, a questionnaire was developed and validated through pilot work and psychometric analysis, to measure the following constructs: knowledge, attitudes, interest, and importance given to biotechnology.

## Purpose of the study

The main goal of this study was to present an oriented guideline for validating scores of quantitative instruments in applied settings, by focusing on the psychometric analysis of data gathered through the large-scale validation of a multi-dimensional questionnaire designed to measure elementary and high school students’ perceptions about biotechnology. The procedure conducted followed a statistical approach combining a pilot study and psychometric analysis through EFA and reliability analysis. More than produce a valid instrument, this study discusses key issues that are determining for the improvement of the validity and reliability of quantitative survey data through exploratory factor analysis, such as:

Deciding on the quantity of data to use and how to address missing values. After determining the sample size, the researcher must select the method(s) to treat missing data (Blalock et al. 2008).

Deciding on a confirmatory or exploratory technique. The development of new instruments and/ or the absence of a robust theoretical model supporting the instrument used, require the use of exploratory techniques (Worthington and Whittaker 2006).

Determining the fitness of the data to factor analysis. The researcher must always assess the factorability of the data set (Worthington and Whittaker 2006).

Deciding on how many factors and items to retain. The researcher must decide on the number of factors emerging from the analysis that explain the maximum amount of variance in the entire set of items, and the number of items that contribute effectively for those factors (Hayton et al. 2004Hogarty et al. 2005).

Assessing a scale’s reliability. The extent to which the variance in the results can be attributed to the latent variables identified must be assessed (DeVellis 2003).

**Figure 1:**
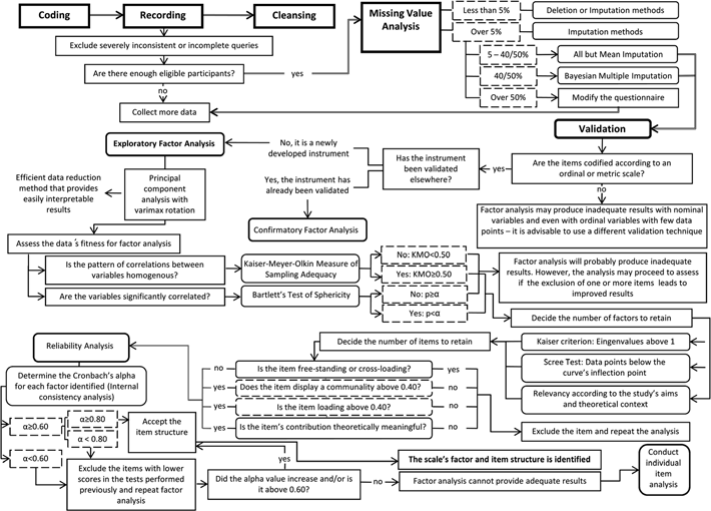
**Methodological workflow proposed for the validation of data from newly developed quantitative assessment instruments through psychometric analysis, using exploratory psychometric analysis (principal component analysis with varimax rotation).**

The guideline proposed to address these topics is emphasized in Figure 1.

## Method

### Participants

This study involved 1196 students attending the 9^th^ grade (aged 14–15 years; *n* = 498) and the 12^th^ grade (aged 17–18 years; *n* = 698) in nine randomly selected schools located in Porto metropolitan area. Students from these instructional levels were asked to participate in this study because these are, respectively, the final years of elementary and high school in Portugal, meaning that many students end these school cycles without further formal science education. The Portuguese high school curricular formats were considered for the inclusion of three subsets of high school students: (i) science students that were attending biology (*n* = 210); (ii) science students that were not attending biology (*n* = 225); and (iii) students engaged in non-science courses, such as arts, economics, informatics, or literature (*n* = 263). The 9^th^ graders’ (56% females) mean age was 14.34 (*SD* = 0.66) and the 12^th^ graders’ (53% females) mean age was 17.36 (*SD* = 0.66).

### Measurement instrument

#### Questionnaire development

The questionnaire was designed following a multistep approach comprising the steps listed below.

*Content definition*. The content covered in the questionnaire was defined based on a review of the literature on assessment of student awareness about biotechnology (namely Cavanagh et al. 2004Dawson 2007, Klop and Severiens, 2007Prokop et al. 2007) with the purpose of identifying existing surveys and topics requiring further analysis. Three criteria were considered: i) authenticity - the contents are contextualized in the elementary and high school science curricula (Departamento do Ensino Básico 2001; Direcção-Geral de Inovação e Desenvolvimento Curricular , Direcção-Geral de Inovação e Desenvolvimento Curricular Direcção-Geral de Inovação e Desenvolvimento Curricular 2004), and address issues frequently discussed in the media and in informal science education contexts; ii) intelligibility - the contents are accessible to students from different instructional levels and courses; iii) multidimensionality - the contents comprise diverse elements that prompt conceptual, cognitive, affective, behavioral and motivational engagement.

*Item pool selection*. The item pool was drafted by selecting items available in published studies (such as Dawson 2007Dawson and Soames 2006Gaskell et al. 2006Prokop et al. 2007) that were relevant for the measurement of the dimensions intended in light of the theoretical framework defined, and adapting them according to the specificities of the study sample. Minimum sets of the most informative items were included in the questionnaire to improve its time and cost efficiency, by reducing its length while maintaining internal checks (Oppenheim 1992).

*Expert review*. A preliminary version of the questionnaire was subjected to the scrutiny of two biology teachers, one microbiologist and one psychology researcher, to obtain feedback on content and face validity, and on the accuracy, intelligibility, adequacy and organization of the items. The questionnaire’s re-structuring heralded by this review resulted in the pilot version.

*Pilot study and psychometric analysis of the pilot data*. From October to December 2008, the pilot version of the questionnaire was administered to 92 elementary and high school students from four classes in three of the nine schools involved in the main study. The composition of the pilot sample was consistent with the main sample and included students from one 9^th^ grade class, from one 12^th^ grade biology class and from two 12^th^ grade non-biology classes. Using the Statistical Package for the Social Sciences (SPSS) v. 17.0, the data conveyed was subjected to EFA and to reliability analysis following the procedures described for the large-scale assessment study. EFA results led to the removal of three items and to the revision of several others. A detailed description of the pilot study is available in Fonseca et al. (2009).

#### Questionnaire description

Following the psychometric analysis of the pilot data, a final version of the questionnaire was obtained (Electronic Additional file 1: Table S1). Part I of the instrument includes factual data questions to determine the students’ socio-demographic profile. Part II includes sections assessing knowledge, attitudes and interest about biotechnology, and the importance given to it.

The *knowledge* section includes: a multiple choice question asking students for a definition of biotechnology (Q1); a list of options from which they must select the biotechnology applications they know (Q2); and a True or False question addressing basic aspects about biotechnology applications (Q3), which includes a *Don’t know* option to reduce the social desirability bias (Black, 1999). The *attitudes* section includes 25 five-point Likert-type items organized in three scales according to the tripartite attitude model (Klop and Severiens 2007Rosenberg and Hovland 1960). The *cognitive component* scale (Q5) evaluates students’ approval of different biotechnology applications. The *affective component* scale (Q6 except Q6c) assesses students’ feelings about human embryo research, genetically modified (GM) food labelling, and the capacity to control the consumption of GM foods. The *behavioral component* scale (Q7 and Q11) assesses students’ intention to buy GM products, and to allow access to their genetic information. The *interest* section includes a five point Likert-type scale (Q8 and Q9) measuring students’ interest directly and considering the frequency with which they are actively or passively exposed to information about biotechnology. The *importance* section consists in a five-point Likert type scale (Q4 and Q6c) measuring the importance students attribute to biotechnology in general and to the future impact of biomedical applications.

#### Data collection and analyses

The fieldwork was conducted from January to April 2009, by administering the questionnaire in the respondents’ native language, during classes under the supervision of a teacher and/or a researcher. From the 1244 students originally asked to participate in the study, 48 had to be excluded as their answers were severely incomplete or inconsistent. Using SPSS v. 17.0, the data collected from 1196 students was codified, recorded and cleansed. Descriptive and missing values analyses were performed for all the items in the questionnaire, followed by validity and reliability analyses.

*Construct validity*. Each of the scales included in the questionnaire was subjected to exploratory factor analysis (principal component analysis with varimax rotation), following the procedures described in Figure 1. The number of factors to retain during factor analysis was decided based on the Kaiser criterion (eigenvalues greater than 1), the scree test and meaningfulness of the results according to the theoretical framework (Costello and Osborne 2005Hayton et al. 2004). The analysis included items that were not freestanding, cross-loading or decreasing the scale’s internal consistency, and that displayed acceptable communalities (above 0.40), with factor pattern/structure coefficients above 0.40 (Costello and Osborne, 2005Fabrigar et al. 1999. Hogarty et al. 2005Sharma 1996). In performing EFA, the Kaiser-Meyer-Olkin (KMO) measure of sampling adequacy and the Bartlett’s Test of Sphericity were used to assess the suitability of the sample for factor analysis (Worthington and Whittaker 2006).

*Reliability*. Following EFA, an internal consistency analysis was performed by determining the Cronbach’s coefficient alpha for each factor identified (DeVellis 2003).

*Cross-validation.* The consistency of these analyses was assessed through cross-validation (Hastie et al. 2009), by repeating the procedures for two independent sub-samples resulting from aleatory bipartition of the main sample and for the four subsets in which it can be divided according to grade and curricular structure: 9^th^ graders, 12^th^ grade science students attending biology; 12^th^ grade science students that were not attending biology; and 12^th^ graders from other courses. The two cross validation sub-samples were obtained by organizing the database according to the different groups that can be defined by the respondents’ grade, course, school and gender and by randomly attributing the code 1 or 2 to half of the individuals of each group.

*Dichotomous items*. The *knowledge* section includes dichotomous items that were not subjected to factor analysis. Decisions on item retention and adequacy regarding these sections were made according to the outcomes of missing values analysis (Kent 2001), and considering the Kuder-Richardson (KR20) coefficient scores. Item difficulty and item discrimination indexes were determined for each item in the knowledge section, allowing to assess the questionnaire’s ability to distinguish between the different academic profile-based groups (Netemeyer et al. 2003).

*Inferential statistics*. The students’ responses were examined and compared by performing Student’s *t*-tests and ANOVA analysis. One-sample *t*-tests were used to compare the students’ mean responses with the midpoint of the test variables (test value = 3). For a confidence interval of 95%, responses that were below, above or equal to 3 were, respectively considered indicative of a negative, positive and neutral positioning. Correlations between variables were assessed using Pearson’s product–moment correlation coefficient.

## Results and discussion

Understanding the psychometric properties of the instruments used in quantitative research is essential in order to make sense of the data they provide. Since this study discusses the implications of psychometric analysis for a comprehensive understanding of the findings according to the theoretical framework and the interactions between the variables, the detailed description of this survey’s outcomes is beyond the focus of this work and can be found in Fonseca et al. (2011).

### Missing values analysis

Following missing values analysis, all items were considered eligible for analysis. The maximum score of missing cases registered was 2.3% and occurred for item Q1 (Electronic Additional file 1: Table S1). The series mean method in SPSS was used to replace the missing values, given that they were limited (below 5%) and random (Paul et al. 2008).

### Knowledge

The Kuder-Richardson 20 (KR20) score for the knowledge section was 0.55. The KR20 formula provides an estimate of internal consistency for inventories with dichotomous items, which is interpreted like the Cronbach alpha scores (Gravetter and Forzano 2009). Ranging from 0.00 to 1.00, KR20 scores must be greater than 0.60 for a measure to be considered reliable (Wasserman and Bracken 2003). However, since the KR20 coefficient provides minimum reliability estimates and the difficulty of the items in this section is heterogeneous (Black, 1999), all items were upheld for analysis. The difficulty of the *knowledge* items varied from 22% to 87%, averaging 49%. The item difficulty index is the fraction of correct answers per item and its optimum value is usually considered to be halfway between 100% of correct answers for that item and the chance probability of getting the answer correct (Kaplan and Saccuzzo 2009). Therefore, the scores obtained indicate the possibility of differentiating several levels of student knowledge. The mean item discrimination value was 0.31, ranging from 0.18 to 0.44, with item Q3a scoring below 0.05. Item discrimination measures the degree of correspondence between the success in each item and in the whole set of items, and can be computed using a point biserial correlation (Kaplan and Saccuzzo 2009). The correlation values must be above 0.30 for items to be considered sufficiently discriminating (Kaplan and Saccuzzo 2009). Although the scores obtained may suggest a weak discriminatory capacity, the item difficulty index together with the ANOVA results for the *Knowledge Score* (0 to 24 points) obtained by combining the selection of the most inclusive option in question Q1 (option ii, Electronic Additional file 1: Table S1), the number of correct answers in Q3 (Electronic Additional file 1: Table S1) and the number of biotechnology applications known by each student (Q2, Electronic Additional file 1: Table S1) demonstrates the questionnaire’s capacity to distinguish between the four academic profile-based groups sampled (*F*(3.1192) = 50.78, *p* < 0.001) (Electronic Additional file 2: Table S2). These results reveal a hierarchical distribution of knowledge according to the biology coverage of each group’s curricula, with the science students who attended biology scoring significantly higher than the other students (*p* < 0.001), followed by the science students who did not attend biology and finally the non-science students and the 9^th^ graders, between whom no significant differences were observed (*p* = 0.40).

### Scales

Considering that the scales in this questionnaire were newly developed, their validation was conducted through EFA (Worthington and Whittaker 2006). From the existing extraction procedures for EFA, principal component analysis (PCA), and common factor analysis are the two most frequently used, and there has been disagreement among statisticians about their advantages and limitations (Costello and Osborne 2005Worthington and Whittaker 2006). In this study, PCA was selected as the extraction method considering that: (i) in most contexts, both methods have been shown to produce similar results (Fabrigar et al. 1999); (ii) PCA is the default option in most statistical software packages, such as SPSS and SAS (Statistical Analysis System), and consequently, more easily available; (iii) compared with PCA, the outcomes of common factor analysis pertain more effectively to confirmatory factor analysis, making it appropriate for studies for which there is not a utterly established theoretical model (Floyd and Widaman 1995). Concerning the rotation method used, both orthogonal and oblique rotation methods were tested, producing identical outcomes regarding factor pattern/structure correlations. Since the interpretation of rotated component matrixes is simpler, it was decided to present the outcomes of varimax rotation (orthogonal) (Costello and Osborne 2005Henson and Roberts 2006).

#### Attitudes’ cognitive component scale

According to the pilot study data, this scale includes three factors: *classical applications* (Q5a and Q5b, *α* = 0.64.); *agro-food applications* (Q5d, Q5i, and Q5j, *α* = 0.62); and *biomedical applications* (Q5h, Q5k, and Q5l, *α* = 0.67). The factor structure identified in the large-scale evaluation is consistent with this three-factor solution (Table 1), and explains 64.47% of the total variance observed. The Kaiser-Meyer-Olkin (KMO) score was 0.80, confirming the sample’s adequacy for factor analysis (KMO = 0.80). The KMO provides a measure of homogeneity between variables, by comparing partial correlations coefficients with the observed correlation coefficients (Worthington and Whittaker 2006), and it should be greater than 0.50 for a satisfactory factor analysis to proceed (Sharma 1996). Furthermore, Barlett’s Test of Sphericity shows a statistically significant correlation between the variables (*χ*^*2*^(28) = 2010.08*, p* < 0.001). This test allows assessing the quality of the correlation matrix, by testing the null hypothesis that it is an identity matrix. Significant scores for the Bartlett’s Test (*p* < 0.05) indicate that there is a correlation pattern among the variables tested (Ho 2006). In addition, the Cronbach’s alpha values are all satisfactory, scoring above 0.60. Cronbach’s alpha provides an average estimate of all possible split-half correlations obtained by dividing a scale in every possible way. Scores vary from 0.00 to 1.00 and must be greater than 0.60 for a measure to be considered reliable (Wasserman and Bracken 2003). Thus, it was decided to keep this factor structure and analyse the data accordingly.

**Table 1:** **Factor structure of the** ***cognitive component of attitudes*** **scale based on EFA and reliability analysis**

		Identifiable factors			One sample ***t***-test(test value=3)			
Item	***h*** ^***2***^	***Classical applications***	***Agro-food applications***	***Biomedical applications***				
					***M***	***SD***	***t***(1195)	***p***
*Q5a. Use of yeast in the production of bread, wine and beer*	0.75	0.85			3.76	1.15	23.04	<0.001
*Q5b. Use of yeast in animal food production*	0.69	0.79			3.33	1.15	10.06	<0.001
*Q5d. Plant growth improvement in saline environments by gene alteration*	0.66		0.81		3.56	1.11	17.56	<0.001
*Q5i. Production of pesticide resistant plants by gene manipulation*	0.66		0.77		3.45	1.24	13.89	<0.001
*Q5j. Genetic modification of tomatoes to make them ripen more slowly and have a longer shelf life*	0.48		0.61		2.74	1.26	−7.18	<0.001
*Q5h. Utilization of genetically modified cows in the production of medicines for humans*	0.58			0.65	2.61	1.20	−11.2	<0.001
*Q5k. Use of insulin produced by bacteria*	0.62			0.71	3.33	1.25	9.13	<0.001
*Q5l. Organ transplant from transgenic animals to humans*	0.71			0.84	2.71	1.31	−7.53	<0.001
Eigenvalue		1.00	3.09	1.06				
% of variance		12.54	38.68	13.25				
Cronbach’s alpha		0.64	0.66	0.67				
	*M*	3.52	3.25	2.90				
	*SD*	0.98	0.90	0.98				
	*t*(1195)	19.32	9.64	−4.08				
	*p*	<0.001	<0.001	<0.001				

EFA - exploratory factor analysis. Coefficients below 0.30 were suppressed. KMO = 0.80. Bartlett’s Test of Sphericity: *χ* ^*2*^(28) = 2010.08*, p* < 0.001. *h* ^*2*^ - communality coefficient. *M* - Mean. *SD* - Standard Deviation.

The factors’ mean scores (Table 1) reveal a hierarchical approval of the three different types of applications considered (*p* < 0.01), with classical applications being considered the most acceptable, followed by agro-food applications, and with biomedical applications being disapproved by the majority of students. This is an unexpected result considering that usually biomedical applications are perceived as more acceptable than agro-food applications (Klop and Severiens 2007; Sáez et al. , Sáez et al. Sáez et al. 2008). Since two of the three items contributing to the factor *biomedical applications* mention animal manipulation (Q5h and Q5l, Table 1), which is known to elicit negative attitudes (Dawson 2007Einsiedel 2005), it is possible that the students’ positioning towards biomedical applications is a response to the type of organism manipulated rather than to the purpose of the application. In fact, individual item analysis shows that the mean scores for both of these items were significantly lower than 3, whereas item Q5k (Table 1), addressing bacterial manipulation, scored significantly above 3 in the five-point scale used. These outcomes demonstrate the impact of item content on students’ responses, and assert the importance of considering the multidimensionality of the variables measured, which would not be evident by conducting a simple reliability analysis of all the items included in the *cognitive component* scale. In this case, the Cronbach alpha value obtained would be satisfactory (α = 0.81) and the global mean score for the scale would be indicative of a positive cognitive appraisal (*M* = 3.27, *SD* = 0.65, *t*(1195) = 14.44, *p* < 0.001). These results would overlook differential attitudinal responses, emphasizing the need to consider a scale’s factor structure as finer information that shapes the global information provided by that scale.

#### Attitudes’ affective component scale

According to the factor structure identified during the pilot data processing, this scale includes two factors: *human embryo research* (Q6a and Q6d, *α* = 0.37); and *control capacity* (Q6b and Q6e, *α* = 0.33). The best factor solution obtained by EFA corroborates this two-factor structure (Table 2) and accounts for 58.73% of the variance observed. However, these results are not supported by the reliability analysis, as the factors included in the scale do not display acceptable internal consistency (scoring below 0.50*)*. Moreover, despite the statistically significant correlation between the variables (*χ*^*2*^(6) = 142.99*, p* < 0.001), the KMO index (KMO = 0.49) indicates a disperse pattern of correlations among them (Sharma 1996), suggesting that the items’ formulation leads this dimension to be unfit for factor analysis. In fact, the KMO score obtained for the pilot sample (KMO = 0.45) was also at the threshold of acceptability, suggesting that the increase in the sample size does not affect its suitability for this method. Whereas these results may be interpreted as tendencies when a relatively small sample is used, they become unacceptable for a sample of 1196 individuals. Therefore, this factor structure should not be considered. This outcome is not surprising, given the also low internal consistency scores registered during the psychometric analysis of the pilot study’s data. A solution to overcome this situation involves increasing the number of items contributing for the two factors identified (Black 1999Kent 2001).

**Table 2:** **Factor structure of the a** ***ffective component of attitudes*** **scale based on EFA and reliability analysis**

		Identifiable factors		One sample ***t***-test (test value = 3)			
Item	***h*** ^***2***^	***Human embryo research***	***Control capacity***	***M***	***SD***	***t***(1195)	***p***
*Q6a. It is our duty to authorize investigation that may lead to the development of more efficient medical treatments, even if it implies using embryonic stem cells*	0.67	0.80		2.98	1.23	−0.63	0.52
*Q6d. It is wrong to use embryonic stem cells in biomedical research, even if it may contribute to the development of medical treatments (R)*	0.69	0.82		2.98	1.27	−0.55	0.58
*Q6b. The labels of transgenic food should specify whether the food or any of its ingredients is genetically modified*	0.37		0.61	4.62	0.80	69.83	<0.001
*Q6e. Each of us is capable of determining our intake of transgenic foods*	0.63		0.79	3.01	1.22	0.33	0.74
Eigenvalue		1.33	1.02				
% of variance		33.30	25.43				
Cronbach’s alpha		0.48	0.03				
	*M*	3.00	2.19				
	*SD*	1.02	0.74				
	*t*(1195)	−0.04	−37.88				
	*p*	0.97	<0.001				

EFA - exploratory factor analysis. Coefficients below 0.30 were suppressed*.* KMO = 0.49. Bartlett’s Test of Sphericity: *χ* ^*2*^(6) = 142.99*, p* < 0.001. *h* ^*2*^ - communality coefficient. *M* - Mean. *SD* - Standard Deviation. R - reversely coded item.

#### Attitudes’ behavioral component scale

According to the pilot data, this scale has a two-factor structure *- buying intent* (Q7a, Q7b, Q11c, and Q11d), *α* = 0.63) and *access to genetic information* (Q11a and Q11b, *α* = 0.69). The best factor solution identified during the large scale evaluation is consistent with this two-factor scale (Table 3) and explains 61.00% of the total variance observed. The sample adequacy is confirmed by the KMO score (KMO = 0.74) and the Bartlett’s Test of Sphericity demonstrates that the variables are statistically significantly correlated (*χ*^*2*^(15) = 1378.22*, p* < 0.001). However, the Cronbach’s alpha value for the factor *access to genetic information* is below the threshold of acceptability (*α* = 0.56). The individual analysis of the two items that contribute to this factor (Q11a: *M* = 3.35, *SD* = 1.22, *t*(1195) = 9.98, *p* < 0.001; Q11b: *M* = 2.48, *SD* = 1.22, *t*(1195) = −14.69, *p* < 0.001) reveals that the differences in their responses were conspicuous enough to prevent their treatment and interpretation as a single underlying variable.

**Table 3:** **Factor structure of the** ***behavioral component of attitudes*** **scale based on EFA and reliability analysis**

		Identifiable factors		One sample ***t***-test (test value = 3)			
Item	***h*** ^***2***^	***Buying intent***	***Access to genetic information***	***M***	***SD***	***t***(1195)	***p***
*Q7a. Buy transgenic foods if they were easily available in supermarkets*	0.71	0.84		2.78	1.05	−7.17	<0.001
*Q7b. Buy medicines obtained by genetically manipulation*	0.59	0.77		2.93	1.07	−2.24	0.03
*Q11c. Buy transgenic foods if they were healthier than other foods*	0.55	0.57		3.51	1.16	15.28	<0.001
*Q11d. Buy transgenic foods if they were less expensive than other foods*	0.49	0.67		2.53	1.21	−13.55	<0.001
*Q11a. Do a genetic test for medical diagnosis*	0.67		0.81	3.35	1.22	9.98	<0.001
*Q11b. Give the police access to your genetic information*	0.65		0.80	2.48	1.22	−14.69	<0.001
Eigenvalue		2.51	1.15				
% of variance		41.83	19.17				
Cronbach’s alpha		0.72	0.56				
	*M*	2.94	2.92				
	*SD*	0.83	1.02				
	*t*(1195)	−2.594	−2.83				
	*p*	0.01	0.01				

EFA - exploratory factor analysis. Coefficients below 0.30 were suppressed. KMO = 0.74. Bartlett’s Test of Sphericity: *χ* ^*2*^(15) = 1378.22*, p* < 0.001. *h* ^*2*^ - communality coefficient. *M* - Mean. *SD* - Standard Deviation.

#### Interest about biotechnology scale

EFA results for the pilot data indicate this is a uni-factor scale (Q8, Q9a, Q9b, and Q9c, *α* = 0.77). The large scale evaluation results corroborate this solution (Table 4), which explains 62.90% of the total variance observed. There is a statistically significant correlation between the variables tested (*χ*^*2*^(6) = 1511.78*, p* < 0.001) and the KMO index supports the sample’s adequacy (KMO *=* 0.77). Furthermore, the scale’s reliability (*α* = 0.80) justifies the retention of this factor structure and the analysis of its items. An important feature of this interest scale is the fact that there is only one item inquiring students directly about their interest in biotechnology (Q8, Electronic Additional file 1: Table S1), whereas there are three items assessing the frequency with which they are passively or actively involved in searching information about it (Q9, Electronic Additional file 1: Table S1). This structure allows minimizing the social desirability bias (Black 1999).

**Table 4:** **Factor structure of the** ***interest about biotechnology*** **scale based on EFA and reliability analysis**

		Identifiable factor	One sample ***t***-test (test value = 3)			
Item	***h*** ^***2***^	***Interest about biotechnology***	***M***	***SD***	***t***(1195)	***p***
*Q8. Rate your interest towards biotechnology*	0.54	0.74	3.23	1.09	7.12	<0.001
*Q9a. Listen to news about biotechnology*	0.62	0.79	2.61	1.07	−12.74	<0.001
*Q9b. Read articles or watch TV shows about biotechnology*	0.73	0.85	2.66	1.16	−10.02	<0.001
*Q9c. Search the web for subjects related to biotechnology*	0.66	0.80	2.09	1.10	−28.44	<0.001
Eigenvalue		2.52				
% of variance		62.90				
Cronbach’s alpha		0.80				
	*M*	2.65				
	*SD*	0.88				
	*t*(1195)	13.93				
	*p*	<0.001				

EFA - exploratory factor analysis. Coefficients below 0.30 were suppressed. KMO = 0.77*.* Bartlett’s Test of Sphericity: *χ* ^*2*^(6) = 1511.78*, p* < 0.001*. h* ^*2*^ - communality coefficient. *M* - Mean. *SD* - Standard Deviation.

#### Importance of biotechnology scale

EFA results for this scale with the main study data conform to a uni-factor structure (Table 5) that explains 65.15% of the total variance observed and is consistent with the solution identified using the pilot study data (Q4 and Q6c, *α* = 0.56). However, similarly to what was observed for the *affective component of attitudes* scale, the reliability score does not support this factor solution (*α* = 0.46). Likewise, this scale also seems to be inadequate for factor analysis, given that, although the variables are statistically significantly correlated (*χ*^*2*^(1) = 114.89*, p* < 0.001), the KMO value is at the threshold of acceptability (KMO = 0.50). These results support the need to redefine this scale, by incorporating more items.

**Table 5:** **Factor structure for the** ***importance of biotechnology*** **scale based on EFA and reliability analysis**

		Identifiable factor	One sample ***t***-test (test value=3)			
Item	***h*** ^***2***^	***Importance of biotechnology***	***M***	***SD***	***t***(1195)	***p***
*Q4. How important do you think biotechnology is to the quality of life?*	0.65	0.81	3.75	0.81	32.08	<0.001
*Q6c. Do you agree that future generations will benefit from biotechnology medical applications?*	0.65	0.81	3.99	0.95	36.24	<0.001
Eigenvalue		1.30				
% of variance		65.15				
Cronbach’s alpha		0.46				
	*M*	3.87				
	*SD*	0.71				
	*t*(1195)	42.48				
	*p*	<0.001				

EFA - exploratory factor analysis. Coefficients below 0.30 were suppressed. KMO = 0.50*.* Bartlett’s Test of Sphericity: *χ* ^*2*^(1) = 114.89*, p* < 0.001*. h* ^*2*^ - communality coefficient. *M* - Mean. *SD* - Standard Deviation.

### Articulating EFA results with the theoretical background: interpretation and implications

Most of the instruments used to measure student attitudes towards biotechnology, regardless of the concept definition considered, have envisaged this as a uni-dimensional construct (Dawson and Schibeci 2003Erdogan et al. 2009). Only recently Klop and Severiens (2007) have demonstrated that a tripartite model, underpinned by the interplay between cognitive, affective and behavioral elements, allows a more thorough description of students’ attitudinal responses to biotechnology applications. Consistently with the tripartite model, EFA outcomes using the pilot data and the main study’s data revealed item structures that conform to three different scales. However, this result would not be evident simply by conducting a reliability analysis of all the attitudes items, as the Cronbach alpha value obtained would be satisfactory (α = 0.82). This reasoning applies to each attitude scale defined, and demonstrates that the awareness of a scale’s factor structure enables the researcher to conduct a sounder interpretation of the constructs measured than is achievable through a global appraisal. Considering the tripartite attitude model, although knowledge can exert a positive influence, the development of a certain attitude towards biotechnology relies on emotional and behavioral elements based on personal weighing of risks, benefits, and ethical implications (Brossard and Nisbet 2007). In this study, the different constructs measured were subjected to correlation analyses, whose outcomes were interpreted according to the reference values available in De Vaus (2002): correlations scoring below 0.30, between 0.30 and 0.50, or above 0.50, were, respectively, considered low, moderate or large. Taking this into consideration, knowledge was found to be positively correlated with cognitive and behavioural attitudinal components (*p* < 0.01). The correlations identified between the variables included in each of these domains (Electronic Additional file 3: Table S3) suggest that the development of perceptions about biotechnology applications depends on an intricate network of attitudinal elements that modulate the expression of knowledge (Amin et al. 2007Klop and Severiens 2007; Sáez et al. , Sáez et al. Sáez et al. 2008). For instance, although associated with knowledge (*r* = 0.25, *n* = 1196, *p* < 0.001), the intention to purchase GM products was more strongly correlated with the students’ beliefs about agro-food (*r* = 0.45*, n* = 1196*, p* < 0.001) and biomedical (*r* = 0.46, *n* = 1196, *p* < 0.001) applications. In addition to attitudes, motivational elements are also important determinants of people’s behaviours. Specifically, student interest about biotechnology can be regarded as an endogenous determinant of motivational patterns (Ryan and Deci 2000). EFA results revealed a uni-factor *interest* scale, according to which students were not that interested about biotechnology (*M* = 2.65, *SD* = 0.88, *t*(1195) = 13.93, *p* < 0.001). In addition, this dimension was positively correlated with the students’ *Knowledge Score* (*r* = 0.36, *n* = 1196, *p* < 0.001). Future research on the correlation patterns identified between the dimensions measured and those requiring further development, i.e. the affective component of attitudes and student interest about biotechnology, will foster their transposition into causal relationships, informing practitioners and curriculum developers of the most efficient interventional measures.

### Cross validation

The outcomes of the cross validation procedures confirm the results obtained using the main student sample (Electronic Additional file 4: Table S4 and Additional file 5: Table S5). The best solutions identified for the two subsets obtained by aleatory division of the main sample were straightforward asserting. When the sample was divided into four groups according to the student’s grade and curricular structure, the variations of the scales’ internal consistency demanded a more careful and adjusted selection of the number of factors and items to retain. To a certain extent this variability could be predicted considering the heterogeneity of the participants’ academic profiles among these four groups.

Overall, the factor structures for each of the scales in the questionnaire identified during the large-scale evaluation (Tables 1, 2, 3, 4 and 5) are consistent with the ones previously identified with the pilot sample (*n* = 92), and sustained by the cross-validation procedures (Electronic Additional file 4: Table S4 and Additional file 5: Table S5). Although the increase in the number of respondents might have been expected to produce a sounder factor structure, this was not observed. Furthermore, EFA results were only partially supported by the outcomes of the reliability analysis. Consequently, the factor structures that showed poor reliability should not be upheld.

Although there is not a specific procedure to determine the adequate sample size for EFA (Reise et al. 2000), criteria such as keeping a minimum 2:1 ratio between the number of subjects and variables assessed (Meyers et al. 2006), or having a minimum of 100 effective participants (Child 2006Wasserman and Bracken 2003) are considered reasonable. Nevertheless, regardless of the criteria considered, samples must be sufficiently large to allow minimizing sampling errors, obtaining stable estimates, and producing robust statistical analyses (Wasserman and Bracken 2003). This study’s findings corroborate the premise that sample adequacy for EFA rests on practical decisions concerning desired levels of statistical significance and meaningful analytic contributions for the theoretical aims of the study (Costello and Osborne 2005Henson and Roberts 2006). Furthermore, they emphasize that the EFA’s efficiency depends not only on the number of individuals providing the data (Costello and Osborne 2005Fabrigar et al. 1999), but also on their homogeneity concerning features such as age or education. In addition, these outcomes also indicate that even with representative samples, extrapolations must be cautious and rational (Child 2006). The sample used in this study was heterogeneous in regards to the participants’ academic background. Although cross validation using the four key groups that compose the main sample ultimately confirmed the best factor structures identified, the variability of the results obtained between groups suggests that the sample’s composition is clearly a conditioning factor. This heterogeneity can also explain why the factor structures identified were not as clear as what might be expected considering the size of the sample (Child 2006Costello and Osborne 2005). It is possible that these scores are a consequence of the low number of items contributing to the factors considered. Yet, increasing the number of items may not always be an option, as a longer questionnaire can be inappropriate for specific target populations. For instance, presenting students within the age range of this study’s participants with a longer questionnaire is likely to foster their impatience, resulting in their lack of engagement, which would jeopardize the reliability of their answers (Dörnyei 2002Oppenheim 1992).

It is necessary to keep in mind that EFA is an approach that requires adopting a pragmatic position and deciding upon the articulation of results that frequently do not fit perfectly into the existing criteria (Fabrigar et al. 1999). Therefore, its outcomes are influenced by several factors, namely the design of the study, its aims or the data properties (Costello and Osborne 2005). For example, even if a more parsimonious interpretation of the data is achievable through factor analysis, it is imperative to appraise its results according to the theoretical framework in which the study was designed (Hayton et al. 2004). Practical decisions that affect the efficiency of EFA, such as factor and item retention or the size of the sample to be used, cannot be devoid of theory. Despite its limitations, this statistical method is a powerful tool to identify latent variables that can account for the variance observed for certain psychological features, to better understand the correlations between the variables, and to integrate the results obtained within theoretical constructs (Henson and Roberts 2006). In this study, EFA proved to be utmost important in allowing to focus the data analysis on underlying constructs that were not obvious in the original design, in improving the interpretation of the results according to the existing theoretical frameworks and in bringing to the fore a multidimensional characterization of students perceptions about biotechnology according to key psychometric features.

## Conclusions and Implications for science education research

This study reinforces the notion that the forecasts made available by EFA are affected by sample size and composition, and that the use of larger samples does not necessarily yield better results (Hogarty et al. 2005Reise et al. 2000). Most importantly, it demonstrates that the decisions required in psychometric analysis are not straightforward and depend on the nature of the study design, the goals set, and the properties of the data collected.

The questionnaire used in this study allows obtaining a broad characterization of elementary and high school students’ perceptions about biotechnology with reasonable validity and reliability. Furthermore, because the samples used in the pilot and in the large-scale assessment study comprise students from diverse academic backgrounds within a wide age range, this questionnaire is suitable for implementation in a variety of instructional situations and contexts. In addition, by allowing to collect data on various elements in a single application, this instrument is a time-efficient option even for studies with tight research agendas. However, more than present the science education research community with a novel quantitative measurement instrument, this work contributes with the definition of a procedural guideline for studies framed within the scope of quantitative assessment that can be applied to the improvement of the validity and reliability of the data collected in diverse evaluative settings. In this context, it must be mentioned that this study does not seek to produce a better or generally more suitable instrument than the biotechnology knowledge inventories and attitudes scales available in published research. Likewise, the validation procedure presented is not exclusive nor to be applied in every science education survey developmental study. The goal is to provide an insightful perspective on an efficient and easily available validation procedure that has wide applicability in quantitative research. It contributes to demonstrate that psychometric analysis methods are not impervious statistical techniques that may seem unappealing and complex to unacquainted researchers.

As future research, it would be interesting to apply the questionnaire in different countries to assess public perceptions about biotechnology in both student and adult populations. Since it covers general topics that are not exclusively curricular-derived, the questionnaire can be used with populations from various backgrounds. It is possible to further develop the instrument, namely by increasing the number of items for the factors with low internal consistency scores, so that its reliability can be improved. According to the features of the target population and the research plan, the various scales comprised in this larger questionnaire could be administered separately, or in differently combined fashions. In fact, the *attitudes* scales were used in a study focusing on biology teachers’ beliefs about biotechnology and biotechnology education (Fonseca et al. 2012). In addition, the data of the large-scale implementation study presented (Fonseca et al. 2011) has been applied to the development of a hands-on activity to improve high school students’ scientific literacy (Fonseca and Tavares 2011). The instrument’s multi-dimensional features fostered the cross-examination of the dimensions evaluated in order to design the most suitable experimental activities, namely concerning criteria such as authenticity, intelligibility, motivation and engagement. Finally, it would be important for other researchers to implement this proposed guideline using their own instruments and datasets at pilot or post-implementation stages. By allowing them to scrutinize their data, this will give them a deeper understanding of their quality and intricacies, thus improving the results and the generalizations made.

## Electronic supplementary material

Additional file 1: Table S1: Questionnaire used. (DOCX 22 KB)

Additional file 2: Table S2: Students’ knowledge about biotechnology. Percentage of students selecting the broadest option in question Q1, mean number of applications listed in Q2 known by students, mean number of correct answers in question Q3, and mean knowledge score values. (DOC 42 KB)

Additional file 3: Table S3: Pearson product-moment correlations between knowledge and attitudes towards biotechnology (*n*=1196). (DOC 49 KB)

Additional file 4: Table S4: Cross validation results using two aleatory sub-samples. (DOC 38 KB)

Additional file 5: Table S5: Cross validation results using 4 sub-samples: 9^th^ graders; 12^th^ grade science students attending biology; 12^th^ grade science students that are not attending biology; and 12^th^ graders from other courses. (DOC 54 KB)
