# Supplementary material for: A statistical approach to quantitative data validation focused on the assessment of students’ perceptions about biotechnology
Source: Springerplus. 2013 Oct 1;2:496. doi: 10.1186/2193-1801-2-496 (PMC3795879; doi:10.1186/2193-1801-2-496)
Supplement: Supplementary file 1 — Additional file 1: Table S1: Questionnaire used. (DOCX 22 KB) [file 40064_2013_568_MOESM1_ESM.docx]

Table S1

.Questionnaire used

| Section / Scale | | Examples of questions/ items |
| --- | --- | --- |
| Factual questions School you are attending; Grade you are attending; Age; Sex; Course you are attending | | |
| Knowledge | Q1: Biotechnology can be defined as a set of processes… (Select the option you most agree with)  (i)… in which recombinant DNA technology is used; (ii)… applied to investigation and product development; (iii)… which involves cell and tissue culture; (iv)… by which genetically modified organisms (GMOs) can be developed.  Q2: From the biotechnological applications listed, select the one(s) you know.  (i) Production of medicines and vaccines; (ii) Production of hormones; (iii) Production of organic products, such as milk or yogurt; (iv) Recovery of contaminated soils using genetically modified bacteria; ... (viii) Production of amminoacids and vitamins.  Q3: Answer the following questions using True/False/I don’t know.  (a) All biotechnology applications are properly regulated; (b) The ingestion of GM foods can induce gene alterations; (c) There is evidence that GMOs can endanger the environment; (d) It is impossible to transfer genes from plants to animals; (e) Biotechnology allows transferring organs from GM animals to humans; (f) Cloning and genetic engineering are similar biotechnology processes; ... (o) Genetic manipulation techniques can allow to increase animal resistance to disease. | |
| Importance | Q4: How important do you think biotechnology is to the quality of life? (1-Not at all important to 5-Very important)^a^  Q6c: Rate your agreement with the following sentence (1-I disagree completely to 5-I agree completely): Do you agree that future generations will benefit from biotechnology medical applications?^a^ | |
| Attitudes – cognitive component | Q5: Rate your approval towards the following activities (1-I do not approve it at all to 5-I approve it completely).  (a)Use of yeast in the production of bread, wine and beer^b^; (b)Use of yeast in animal food production^b^; (c)Use of genetically modified organisms in waste treatment; (d)Plant growth improvement in saline environments by gene alteration^c^; (e)Treatment of genetic disorders by embryonic gene manipulation; (f)Treatment of genetic disorders by human gene manipulation; (g)Insertion of plant genes into animals; (h)Utilization of genetically modified cows in the production of medicines for humans^d^; (i)Production of pesticide resistant plants by gene manipulation^c^; (j)Genetic modification of tomatoes to make them ripen more slowly and have a longer shelf life^c^; (k)Use of insulin produced by bacteria^d^; (l)Organ transplant from transgenic animals to humans^d^; (m)Use of human cloning with therapeutic purposes. | |
| Attitudes – affective component | Q6: Rate your agreement with the following sentences (1-I totally disagree to 5-I totally agree).  (a)It is our duty to authorize investigation that may lead to the development of more efficient medical treatments, even if it implies using embryonic stem cells^e^; (b)The labels of transgenic food should specify whether the food or any of its ingredients is genetically modified^f^; (d)It is wrong to use embryonic stem cells in biomedical research, even if it may contribute to the development of medical treatments^e^; (e)Each of us is capable of determining our intake of transgenic foods^f^. | |
| Attitudes – behavioral component | Q7: How often would you… (1-Never to 5-Always)  (a)...buy transgenic foods if they were easily available in supermarkets^g^; (b)...buy medicines obtained by genetically manipulation^g^.  Q11: How often would you… (1-Never to 5-Always)  (a)...do a genetic test for medical diagnosis^h^; (b)...give the police access to your genetic information^h^; (c)...buy transgenic foods if they were healthier than other foods^g^; (d)...buy transgenic foods if they were less expensive than other foods^g^. | |
| Interest | Q8: Rate your interest towards biotechnology (1-I am not interested at all to 5-I am very interested)^i^.  Q9: How often do you… (1-Never to 5-Many times)  (a)…listen to news about biotechnology^i^; (b)…read articles or watch TV shows about technology^i^; (c)…search the web for subjects related to biotechnology^i^. | |

The items identified with the same letter (a,b,…i) contribute to the same factor. The questionnaire includes two items, asking students to evaluate their understanding of news about biotechnology and inquiring about the interest of the questionnaire, that were not considered in the analysis. It also includes two questions about use and trust in information sources that were not considered for the purpose of this study. The full version is available upon request from the authors.
