# Supplementary material for: A statistical approach to quantitative data validation focused on the assessment of students’ perceptions about biotechnology
Source: Springerplus. 2013 Oct 1;2:496. doi: 10.1186/2193-1801-2-496 (PMC3795879; doi:10.1186/2193-1801-2-496)
Supplement: Supplementary file 2 — Additional file 2: Table S2: Students’ knowledge about biotechnology. Percentage of students selecting the broadest option in question Q1, mean number of applications listed in Q2 known by students, mean number of correct answers in question Q3, and mean knowledge score values. (DOC 42 KB) [file 40064_2013_568_MOESM2_ESM.doc]

Table S2

Students’ knowledge about biotechnology. Percentage of students selecting the broadest option in question Q1, mean number of applications listed in Q2 known by students, mean number of correct answers in question Q3, and mean knowledge score values

|  | Total | 12OC | 12NB | 12B | 9 | *F*(3,1992) | *p* |
| --- | --- | --- | --- | --- | --- | --- | --- |
| Q1 |  |  |  |  |  |  |  |
| Q1.Option ii | 31.30% | 21.30% | 38.20% | 53.30% | 24.10% |  |  |
| Q1.Other options | 66.40% | 77.60% | 58.10% | 45.70% | 73.00% |  |  |
| Q1.DK/NA | 2.30% | 1.10% | 3.60% | 1,00% | 3.00% |  |  |
| Q2 |  |  |  |  |  |  |  |
| *M* | 4.15 | 3.95^b^ | 4.39^a,b^ | 4.50^a^ | 4.00^b^ | 6.62 | <0.001 |
| *SD* | 1.75 | 1.95 | 1.58 | 1.58 | 1.67 |  |  |
| Q3 |  |  |  |  |  |  |  |
| *M* | 6.98 | 6.13^c^ | 7.45^b^ | 8.55^a^ | 6.44^c^ | 44.53 | <0.001 |
| *SD* | 2.62 | 2.63 | 2.71 | 2.28 | 2.38 |  |  |
| Knowledge Score |  |  |  |  |  |  |  |
| *M* | 11.44 | 10.48^c^ | 12.22^b^ | 13.58^a^ | 10.69^c^ | 50.78 | <0.001 |
| *SD* | 3.47 | 3.47 | 3.54 | 3.02 | 3.13 |  |  |

12OC - 12^th^ graders from non-science courses. 12NB - 12^th^ grade science students not attending biology. 12B - 12^th^ grade science students attending biology. 9 - 9^th^ graders. DK/NA - *Don’t know*/No answer. *M* - Mean. *SD* - Standard Deviation. a, b, c – different letters indicate significant differences for *α*=0.05. See Table 1 for the full items’ description.
