# Supplementary material for: A statistical approach to quantitative data validation focused on the assessment of students’ perceptions about biotechnology
Source: Springerplus. 2013 Oct 1;2:496. doi: 10.1186/2193-1801-2-496 (PMC3795879; doi:10.1186/2193-1801-2-496)
Supplement: Supplementary file 3 — Additional file 3: Table S3: Pearson product-moment correlations between knowledge and attitudes towards biotechnology (n=1196). (DOC 49 KB) [file 40064_2013_568_MOESM3_ESM.doc]

Table S3

Pearson product-moment correlations between knowledge and attitudes towards biotechnology (*n*=1196)

|  | ATCOGII | ATCOGIII | ATCOGIV | ATCOGV | ATCOGVI | ATCOGVII | ATCOGVIII | ATBEHAI | Knowledge |
| --- | --- | --- | --- | --- | --- | --- | --- | --- | --- |
| Classical applications (ATCOGI) | 0.39** | 0.36** | 0.29** | 0.08** | 0.19** | 0.22** | 0.01 | 0.27** | 0.29** |
|  |
| Agro-food applications (ATCOGII) |  | 0.46** | 0.37** | 0.28** | 0.30** | 0.33** | 0.17** | 0.45** | 0.25** |
|  |  |
| Biomedical applications (ATCOGIII) |  |  | 0.41** | 0.22** | 0.26** | 0.47** | 0.27** | 0.46** | 0.36** |
|  |  |  |
| Use of GM microorganisms in waste treatment (ATCOGIV) |  |  |  | 0.24** | 0.27** | 0.17** | 0.11** | 0.31** | 0.28** |
|  |  |  |  |
| Therapeutic embryonic gene manipulation (ATCOGV) |  |  |  |  | 0.59** | 0.17** | 0.25** | 0.27** | 0.12** |
|  |  |  |  |  |
| Therapeutic human gene manipulation (ATCOGVI) |  |  |  |  |  | 0.20** | 0.24** | 0.32** | 0.19** |
|  |  |  |  |  |  |
| Insertion of plant genes into animals (ATCOGVII) |  |  |  |  |  |  | 0.25** | 0.32** | 0.15** |
|  |  |  |  |  |  |  |
| Therapeutic human cloning (ATCOGVIII) |  |  |  |  |  |  |  | 0.22** | 0.01 |
|  |  |  |  |  |  |  |  |
| Intention to purchase GM products (ATBEHAI) |  |  |  |  |  |  |  |  | 0.25** |
|  |  |  |  |  |  |  |  |  |

* indicates significant differences for *α*=0.05; ** indicates significant differences for *α*=0.01.
