# Supplementary material for: A statistical approach to quantitative data validation focused on the assessment of students’ perceptions about biotechnology
Source: Springerplus. 2013 Oct 1;2:496. doi: 10.1186/2193-1801-2-496 (PMC3795879; doi:10.1186/2193-1801-2-496)
Supplement: Supplementary file 4 — Additional file 4: Table S4: Cross validation results using two aleatory sub-samples. (DOC 38 KB) [file 40064_2013_568_MOESM4_ESM.doc]

Table S4

Cross validation results using two aleatory sub-samples

|  |  | Sub-sample 1 | | | | Sub-sample 2 | | | |
| --- | --- | --- | --- | --- | --- | --- | --- | --- | --- |
| Scale | Identifiable factors | KMO | Eigenvalue | % Variance | Cronbach’s alpha | KMO | Eigenvalue | % Variance | Cronbach’s alpha |
| Attitudes  Cognitive component | Classical applications | 0.77 | 1.02 | 12.72 | 0.59 | 0.81 | 1.01 | 12.57 | 0.68 |
|  | Agro-food applications |  | 2.98 | 37.21 | 0.64 |  | 3.16 | 39.50 | 0.67 |
|  | Biomedical applications |  | 1.09 | 13.64 | 0.69 |  | 1.04 | 13.03 | 0.65 |
| Affective component | Human embryo research | 0.46 | 1.34 | 33.44 | 0.50 | 0.51 | 1.33 | 33.24 | 0.46 |
|  | Control capacity |  | 1.08 | 26.88 | 0.08 |  | 1.01 | 25.28 | 0.03 |
| Behavioural component | Buying intent | 0.71 | 2.41 | 40.12 | 0.70 | 0.75 | 2.61 | 43.42 | 0.73 |
|  | Access to genetic information |  | 1.17 | 59.54 | 0.53 |  | 1.13 | 18.79 | 0.58 |
| Interest |  | 0.77 | 2.48 | 62.11 | 0.80 | 0.77 | 2.54 | 63.41 | 0.81 |
| Importance |  | 0.50 | 1.35 | 67.65 | 0.52 | 0.50 | 1.24 | 62.14 | 0.39 |

For simplification purposes, the table does not include information regarding the items that contribute to the factors displayed. The item structure for each factor identified during this analysis is consistent with the one obtained using the main sample. The Bartlett’s Test of Sphericity for each scale is acceptable (*p<*0.001).
