# Supplementary material for: A statistical approach to quantitative data validation focused on the assessment of students’ perceptions about biotechnology
Source: Springerplus. 2013 Oct 1;2:496. doi: 10.1186/2193-1801-2-496 (PMC3795879; doi:10.1186/2193-1801-2-496)
Supplement: Supplementary file 5 — Additional file 5: Table S5: Cross validation results using 4 sub-samples: 9th graders; 12th grade science students attending biology; 12th grade science students that are not attending biology; and 12th graders from other courses. (DOC 54 KB) [file 40064_2013_568_MOESM5_ESM.doc]

Table S5

Cross validation results using 4 sub-samples: 9^th^ graders; 12^th^ grade science students attending biology; 12^th^ grade science students that are not attending biology; and 12^th^ graders from other courses

|  |  | 12^th^ grade science non-biology students | | | | 12^th^ grade science biology students | | | |
| --- | --- | --- | --- | --- | --- | --- | --- | --- | --- |
| Scale | Identifiable factors | KMO | Eigenvalue | % Variance | Cronbach’s alpha | KMO | Eigenvalue | % Variance | Cronbach’s alpha |
| Attitudes’  Cognitive component | Classical applications | 0.77 | 0.91 | 11.35 | 0.52 | 0.79 | 1.03 | 12.85 | 0.52 |
|  | Agro-food applications |  | 3.18 | 39.78 | 0.62 |  | 3.02 | 37.80 | 0.74 |
|  | Biomedical applications |  | 1.23 | 15.36 | 0.74 |  | 1.03 | 12.93 | 0.59 |
| Attitudes’  Affective component | Human embryo research | 0.50 | 1.56 | 38.94 | 0.69 | 0.49 | 1.47 | 36.77 | 0.59 |
|  | Control capacity |  | 1.14 | 28.40 | 0.30 |  | 1.00 | 25.00 | 0.020 |
| Attitudes´  Behavioural component | Buying intent | 0.71 | 2.59 | 43.24 | 0.73 | 0.74 | 2.50 | 41.68 | 0.75 |
|  | Access to genetic information |  | 1.18 | 19.66 | 0.50 |  | 1.19 | 19.75 | 0.50 |
| Interest |  | 0.73 | 2.37 | 59.27 | 0.77 | 0.70 | 2.23 | 5.75 | 0.73 |
| Importance |  | 0.50 | 1.31 | 65.23 | 0.47 | 0.50 | 1.25 | 62.33 | 0.38 |

Table S5 (continued)

|  |  | 12^th^ grade non science students | | | | 9^th^ grade students | | | |
| --- | --- | --- | --- | --- | --- | --- | --- | --- | --- |
| Scale | Identifiable factors | KMO | Eigenvalue | % Variance | Cronbach’s alpha | KMO | Eigenvalue | % Variance | Cronbach’s alpha |
| Attitudes  Cognitive component | Classical applications | 0.71 | 1.04 | 13.05 | 0.75 | 0.73 | 0.99 | 12.40 | 0.51 |
|  | Agro-food applications |  | 1.21 | 15.14 | 0.67 |  | 2.56 | 32.01 | 0.60 |
|  | Biomedical applications |  | 2.91 | 36.31 | 0.60 |  | 1.22 | 15.25 | 0.60 |
| Affective component | Human embryo research | 0.49 | 1.28 | 31.96 | 0.42 | 0.49 | 1.22 | 30.45 | 0.35 |
|  | Control capacity |  | 1.03 | 25.77 | 0.05 |  | 1.06 | 26.46 | 0.08 |
| Behavioural component | Buying intent | 0.75 | 2.60 | 43.26 | 0.71 | 0.70 | 2.36 | 39.34 | 0.68 |
|  | Access to genetic information |  | 1.12 | 18.67 | 0.59 |  | 1.18 | 19.67 | 0.59 |
| Interest |  | 0.76 | 2.45 | 61.22 | 0.79 | 0.78 | 2.48 | 61.98 | 0.80 |
| Importance |  | 0.50 | 1.26 | 63.22 | 0.41 | 0.50 | 1.29 | 64.53 | 0.45 |

For simplification purposes, the table does not include information regarding the items that contribute to the factors displayed. The item structure for each factor identified during this analysis is consistent with the one obtained using the main sample. The Bartlett’s Test of Sphericity for each scale is acceptable (*p*<0.001).
